# Source codes and simulation data for the finite element implementation of the conventional and localizing gradient damage methods in ABAQUS

**DOI:** 10.1016/j.dib.2019.104533

**Published:** 2019-09-18

**Authors:** Subrato Sarkar, I.V. Singh, B.K. Mishra, A.S. Shedbale, L.H. Poh

**Affiliations:** a Department of Mechanical and Industrial Engineering, Indian Institute of Technology Roorkee, Uttarakhand, 247667, India; b Department of Civil and Environmental Engineering, National University of Singapore, 1 Engineering Drive 2, E1A-07-03, 117576, Singapore

**Keywords:** ABAQUS, UEL, UMAT, Subroutine, Source codes, Simulation data, Gradient damage

## Abstract

This data article presents the source codes and obtained simulation data for running numerical fracture simulation in the commercial finite element package, ABAQUS. The computational models implemented through these source codes pertain to the conventional and localizing gradient damage method which are used for the modelling of the fracture phenomena in the components and structures. For a detailed description refer to “A comparative study and ABAQUS Implementation of Conventional and Localizing Gradient Enhanced Damage Models [1]”. The implementation is carried out using a feature in the ABAQUS software called the user defined subroutines. The subroutines are a set of coded files which are used to implement any newly developed computational models depicting actual physical phenomena which are not already available in any commercial software. The user subroutines used in this implementations are UEL and UMAT. The present implementation is very user friendly in the sense that the user needs to just type a couple of commands in the ABAQUS command application to run the simulations. Moreover, the ability of the ABAQUS to run large scale simulations using a very sparse amount of computational resources enables researchers and engineers with limited resources to take advantage of a very advanced computational fracture simulation technique.

**Table:** Specifications Table

Subject area	*Engineering*
More specific subject area	*Computational mechanics*
Type of data	*Source codes and sample simulation data*
How data was acquired	*The source codes and simulation data were generated in-house*
Data format	Input data in (*.txt) format. Code in (*.for) and (*.inp) format. Simulation data in (*.sta), (*.log), (*.msg), (*.dat) and (*.odb) format.
Parameters for data collection	*The simulation data is based on an example problem in Ref*[1]. *The various material and numerical parameters used for the generation of simulation data are mentioned in Section 6.2a of Ref*[1].
Description of data collection	*The simulation data was obtained by running the provided user subroutine code in the commercial FEA package ABAQUS.*
Data source location	*Institution: Indian Institute of Technology* *City: Roorkee* *Country: India*
Data accessibility	*With the article*
Related research article	*Subrato Sarkar, IV Singh, BK Mishra, AS Shedbale, LH Poh.* *A comparative study and ABAQUS implementation of conventional and localizing gradient enhanced damage models* *Finite Elements in Analysis and Design* *10.1016/j.finel.2019.04.001*

**Table:** 

**Value of the data** • The provided source codes are generic in the sense that they can be easily modified and edited to model any kind of material or geometry. • The computational model implemented in the provided source codes and simulation data are used to simulate brittle fracture in elastic solids which can be easily extended to simulate ductile or dynamic fracture. • The present implementation is based on the nonlinear finite element method for a fully coupled system. Hence, the source codes and data could be easily used to model any physical phenomena involving coupled system. • The published source code and data can be used to reproduce the Sarkar et al. [1] paper work.

## 1 Data

The data provided with this article is associated with the computational modelling of gradient damage methods presented in Ref. [1]. The data is segregated into source codes folder and simulation data folder. The source code files are used to provide input data to the ABAQUS solver and run a simulation while the simulation data is obtained after completing the simulation. The provided simulation data is obtained corresponding to a problem in Section 6.2a of paper [1], which simulates failure in a single edge notch specimen. The provided source code folder contains two sub-folders namely **a)** conventional gradient damage model and **b)** localizing gradient damage model. These two sub-folders contain the required files for performing the simulation. The second folder named simulation data contains five files. These files are used to monitor status of the simulation and ensure errorless execution. A detailed description of individual files is provided in a subsequent section. In addition to the source code files and simulation data files, a description on how to run the codes (Fig. 2, Fig. 3, Fig. 4) and visualize simulations (Fig. 5, Fig. 6) in ABAQUS is also presented.

**Fig. 1:**
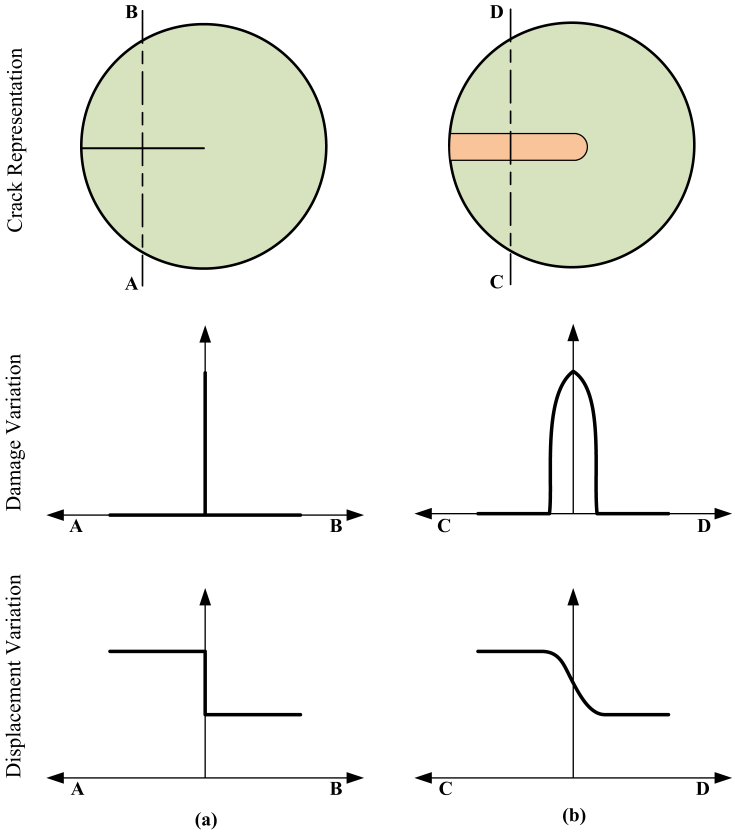
A schematic representation of crack representation, damage variation and displacement variation in **(a)** discrete method and **(b)** smeared method.

**Fig. 2:**
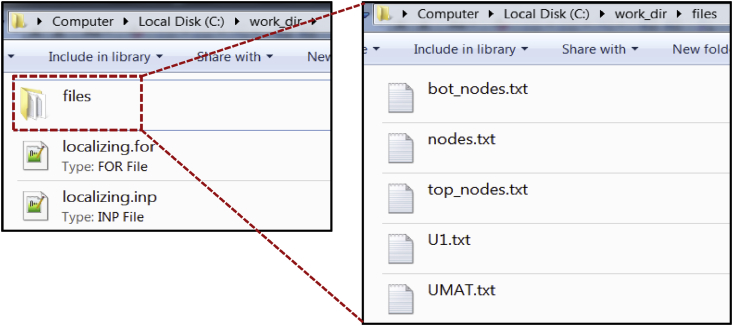
An illustration showing the contents of the work directory viz. the INPUT file, the FORTRAN file and the TEXT files.

**Fig. 3:**
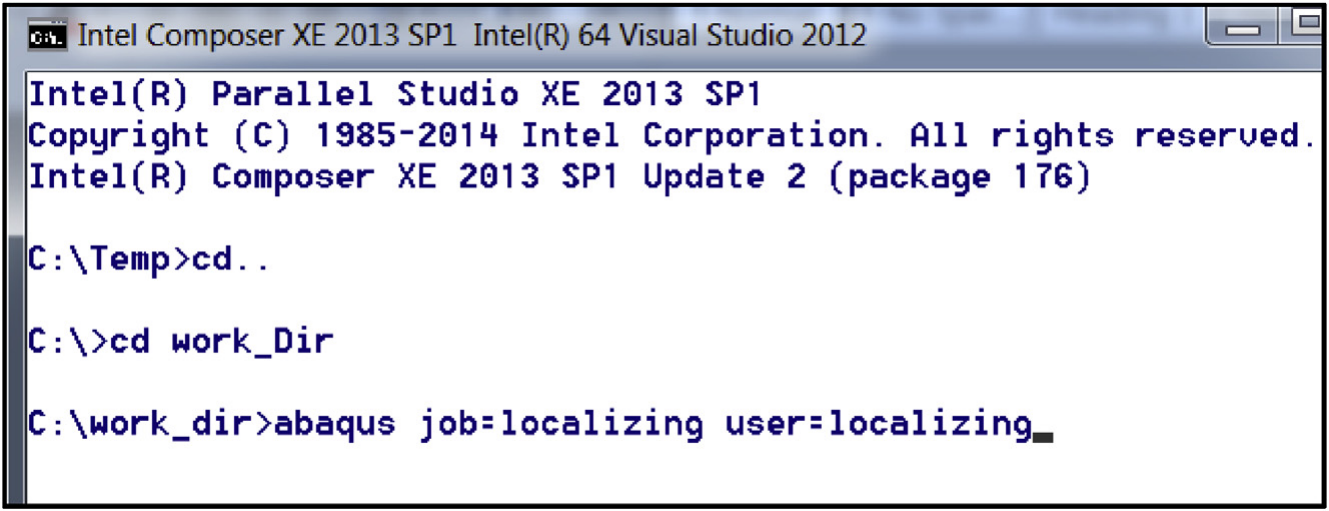
A snapshot of the ABAQUS command application window.

**Fig. 4:**
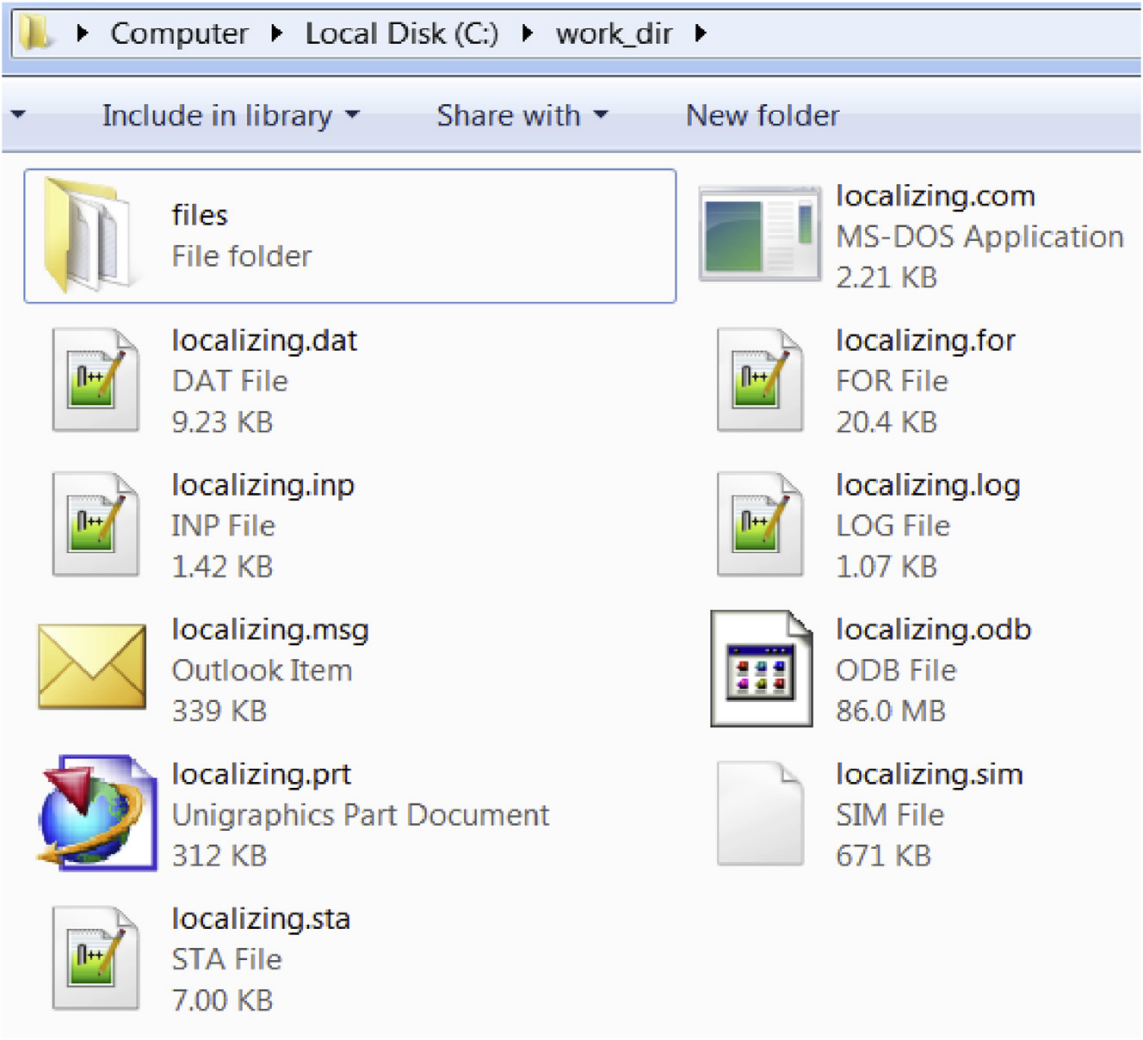
A snapshot of the work directory after the simulation is completed showing the additional files created by ABAQUS solver.

**Fig. 5:**
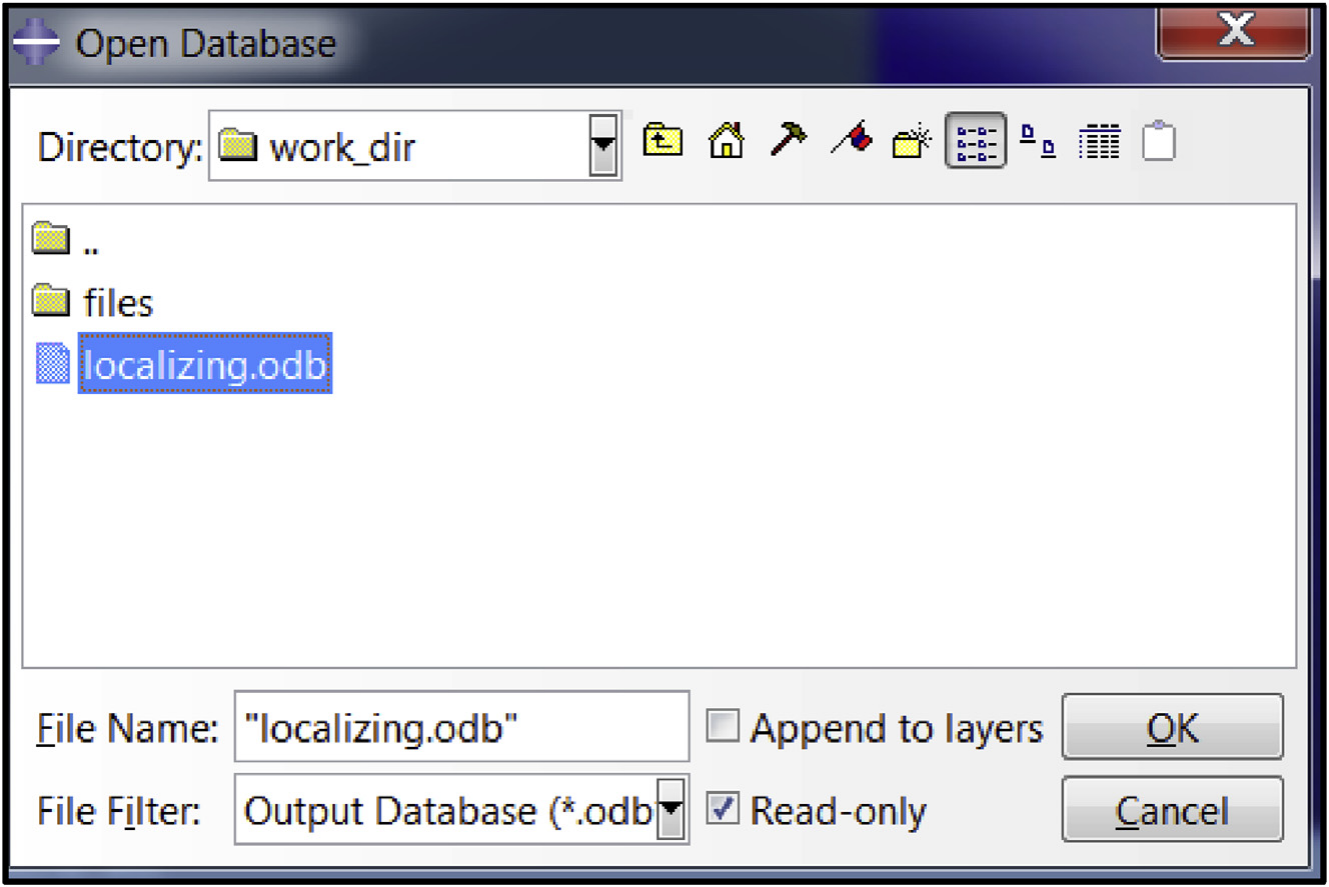
A snapshot of the open database dialogue box in ABAQUS application.

**Fig. 6:**
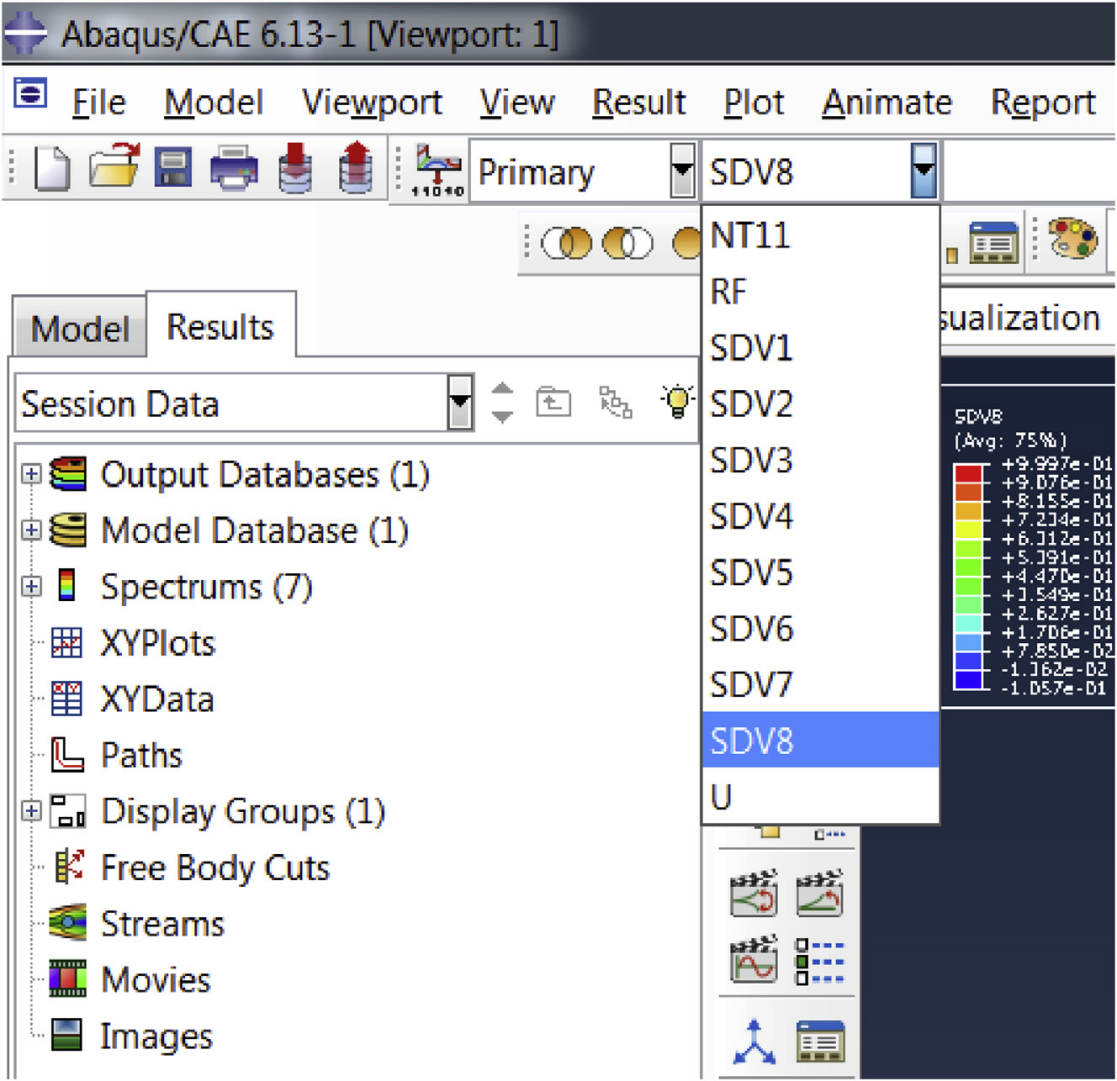
A snapshot of the dropdown menu in the ABAQUS viewer.

## 2 Experimental design, materials, and methods

The gradient damage methods are implemented using the provided source codes and generate simulation data. It must be noted that in order to run the source code files, a user must have a fully installed ABAQUS package along with a FORTRAN compiler and an integrated development environment in the system. For the present implementation, ABAQUS 6.13, Intel Parallel Studio 13 as FORTRAN compiler and Microsoft Visual Studio 12 as IDE have been used.

### 2.1 Gradient damage methods

In the field of computational mechanics, the fracture modelling methods are grouped into two categories i.e. discrete methods and smeared methods. The discrete methods are those in which a discontinuity, like a crack, hole or inclusion, is modelled by a sharp variation in the displacement fields. These sharp variations are incorporated into the displacement fields through enrichment functions. The discrete methods are namely extended FEM [2], [3], [4] extended IGA [5], EFGM [6] to name a few. On the other hand, the smeared methods use a continuous definition of a discontinuity. The gradient damage methods, implemented through the provided data, belong to the smeared methods of fracture modelling. In the gradient damage method, a damage parameter (*D*) is employed to define a crack. This damage parameter is equal to unity in the fully cracked part of the domain and remains zero elsewhere as defined in Eq. (1).

(1) $$D=\left\{ \begin{array}{l} 1\;\text{Completely}\;\text{cracked}\;\text{domain}\\ 0\;\text{Uncracked}\;\text{domain} \end{array}\right.$$

The regions where the damage parameter assumes intermediate values are considered as partially cracked domain. A schematic representation of differences between discrete and smeared crack modelling is shown in Fig. 1.

The gradient damage methods involve nonlinear and coupled field problems. The discretized system of equations for these methods can be expressed in the matrix form as,

(2) $$\left[\begin{array}{cc} K_{uu} & K_{u\bar{\varepsilon }}\\ K_{\bar{\varepsilon }u} & K_{\bar{\varepsilon }\bar{\varepsilon }} \end{array}\right]\left(\begin{array}{c} \delta \underset{˜}{u}\\ \delta \underset{˜}{\bar{\varepsilon }} \end{array}\right)=\left(\begin{array}{c} F^{u}\\ F^{\bar{\varepsilon }} \end{array}\right)$$

where, $K_{uu}$, $K_{u\bar{\varepsilon }}$, $K_{\bar{\varepsilon }u}$ and $K_{\bar{\varepsilon }\bar{\varepsilon }}$ represent the components of the tangent stiffness used in the Newton-Raphson incremental iterative procedure for solving the linear system of equations. $F^{u}$ and $F^{\bar{\varepsilon }}$ are the components of the nodal force vector while $\delta \underset{˜}{u}$ and $\delta \underset{˜}{\bar{\varepsilon }}$ are the obtained incremental solution. For detailed formulation see Ref. [1].

### 2.2 Description of data

The data provided is segregated into two folders:

1. *Source codes*: This folder contains the source codes in the form of ABAQUS input file, FORTRAN file and text files for both the gradient damage models i.e. conventional and localizing models. These files are used by the user to pass information to the ABAQUS solver for a simulation. They are described as,
  a) *ABAQUS input file*: This file contains the input information required by the ABAQUS solver. The input file is written in ASCII format and contains information such as geometry of problem domain, loads, boundary conditions, initial conditions, time increment details, etc.
  b) *FORTRAN file*: This file contains a FORTRAN program which is used to define the computational model for simulating the underlying physics of the fracture phenomenon. The FORTRAN file is written in the form of subroutine which receive and pass variables to the ABAQUS solver.
  c) *Text files*: These are a set of files which are called during the compilation of the input file. These files contain geometric information about the problem domain such as node coordinates, element connectivity and boundary condition node/element sets. These are written in ASCII format.
2. *Simulation data*: A test simulation is carried out to demonstrate the use of provided source codes. The problem is taken from the problem in Section 6.2a in Ref. [1]. The ABAQUS solver generates several files during the simulations. Some of the important files are included in a simulation data folder. The included files are described as,
  a) *Status file* (*.sta): This file shows the status of the simulation during the entire loading history. The status includes the load step number, number of convergence iterations, step time, etc.
  b) *Log file* (*.log): This file logs various operations carried out by the ABAQUS solver such as subroutine compilation, input file reading, simulation completion, etc. In addition to this, the syntax error in the subroutine file is shown in the log file.
  c) *Message file* (*.msg): This file shows various numerical and computational parameters used by the ABAQUS solver such as convergence tolerance, type of linear equation solver, user and CPU time used, etc.
  d) *Data file* (*.dat): This file ensures that the input file has been read successfully and there were no errors encountered during reading input file. It also displays the size of the problem in terms of number of nodes, elements and variables in the model.
  e) *Output database file* (*.odb): This file contains the final output data of the simulation. This file is in hexadecimal format and can only be read by the ABAQUS viewer.

### 2.3 Running the codes

In order to run the source codes, the user must navigate to the work directory. The files in the work directory would look as shown in Fig. 2. The work directory must contain an INPUT file, a FORTRAN file and TEXT files. The TEXT files are kept inside a sub-folder called the “files” which contain the required geometrical data like nodal coordinates and connectivity.

After navigating to the work directory in the ABAQUS command application, type the command: abaqus job = localizing user = localizing. The command application must look like as shown in Fig. 3.

The simulation starts immediately after running the command. There are several files which are created in the work directory by ABAQUS for the monitoring of the simulation (Fig. 4). Some of the important ones are *status file* (*.sta), *log file* (*.log), *message file* (*.msg), *data file* (*.dat) and *output database file* (*.odb). The function and significance of these files have been discussed in the previous section.

### 2.4 Visualization of output data

The *output database file* (*.odb) created by ABAQUS solver during the simulation contains the output data. This database file can only be viewed in the ABAQUS viewer. Hence, in order to view the results, the database file must be opened in the ABAQUS application as shown in Fig. 5.

After opening the database file, the user can easily view the results through dropdown menu on the toolbar as shown in Fig. 6. The nodal variable or the solution dependent variable (SDV) can be selected from the menu as desired by the user.

## References

[1] Sarkar S., Singh I.V., Mishra B.K., Shedbale A.S., Poh L.H. (2019). A comparative study and ABAQUS implementation of conventional and localizing gradient enhanced damage models. Finite Elem. Anal. Des..

[2] Kumar M., Singh I.V., Mishra B.K. (2019). Fatigue crack growth simulations of plastically graded materials using XFEM and J-integral decomposition approach. Eng. Fract. Mech..

[3] Pandey V.B., Singh I.V., Mishra B.K., Ahmad S., Rao A.V., Kumar V. (2019). A new framework based on continuum damage mechanics and XFEM for high cycle fatigue crack growth simulations. Eng. Fract. Mech..

[4] Patil R.U., Singh I.V., Mishra B.K. (2017). A new multiscale XFEM for the elastic properties evaluation of heterogeneous materials. Int. J. Mech. Sci..

[5] Singh S.K., Singh I.V., Mishra B.K., Bhardwaj G. (2019). Analysis of cracked functionally graded material plates using XIGA based on generalized higher-order shear deformation theory. Compos. Struct..

[6] Shedbale A.S., Singh I.V., Mishra B.K. (2016). A coupled FE–EFG approach for modelling crack growth in ductile materials. Fatigue Fract. Eng. M..

